# Industrial point source CO_2_ emission strength estimation with aircraft measurements and dispersion modelling

**DOI:** 10.1007/s10661-018-6531-8

**Published:** 2018-02-22

**Authors:** Federico Carotenuto, Giovanni Gualtieri, Franco Miglietta, Angelo Riccio, Piero Toscano, Georg Wohlfahrt, Beniamino Gioli

**Affiliations:** 1 National Research Council, Institute of Biometeorology (CNR-IBIMET), Via G. Caproni 8, 50145 Florence, Italy; 2 Institute of Ecology, University of Innsbruck, Sternwartestrasse 15, 6020 Innsbruck, Austria; 3 Department of Sciences and Technologies, University of Naples “Parthenope”, Centro Direzionale Isola C4, 80143 Naples, Italy

**Keywords:** Aircraft measurements, Mass balance, WRF/CALMET/CALPUFF models, Emission inventories, Industrial point source

## Abstract

CO_2_ remains the greenhouse gas that contributes most to anthropogenic global warming, and the evaluation of its emissions is of major interest to both research and regulatory purposes. Emission inventories generally provide quite reliable estimates of CO_2_ emissions. However, because of intrinsic uncertainties associated with these estimates, it is of great importance to validate emission inventories against independent estimates. This paper describes an integrated approach combining aircraft measurements and a puff dispersion modelling framework by considering a CO_2_ industrial point source, located in Biganos, France. CO_2_ density measurements were obtained by applying the mass balance method, while CO_2_ emission estimates were derived by implementing the CALMET/CALPUFF model chain. For the latter, three meteorological initializations were used: (i) WRF-modelled outputs initialized by ECMWF reanalyses; (ii) WRF-modelled outputs initialized by CFSR reanalyses and (iii) local in situ observations. Governmental inventorial data were used as reference for all applications. The strengths and weaknesses of the different approaches and how they affect emission estimation uncertainty were investigated. The mass balance based on aircraft measurements was quite succesful in capturing the point source emission strength (at worst with a 16% bias), while the accuracy of the dispersion modelling, markedly when using ECMWF initialization through the WRF model, was only slightly lower (estimation with an 18% bias). The analysis will help in highlighting some methodological best practices that can be used as guidelines for future experiments.

## Introduction

Carbon dioxide (CO_2_) is the primary greenhouse gas (GHG) emitted through human activities (National Research Council 2010). CO_2_ is naturally present in the atmosphere as part of the Earth’s carbon cycle. Human activities have altered the carbon cycle both by adding more CO_2_ to the atmosphere and by influencing the ability of natural sinks, such as forests, to remove CO_2_ from the atmosphere. While CO_2_ emissions come from a variety of natural sources, human-related emissions are responsible for the increase that has occurred in the atmosphere since the industrial revolution. The main human activity that emits CO_2_ is the combustion of fossil fuels (coal, natural gas and oil) for energy and transportation, although certain industrial processes and land use changes also emit CO_2_ (Metz et al. 2007).

Accurate, consistent and internationally comparable data on GHG emissions are essential for the international community to take the most appropriate actions to mitigate climate change, and ultimately to comply with international regulations (United Nations 1998, 2006; De Boer 2008). Communicating relevant information on the most effective actions to reduce emissions and adapt to the adverse effects of climate change also contributes towards global sustainable development. The European regulation (European Parliament and European Council 2013) implements the obligation to compile yearly inventories of GHG emissions at national scale. Emission inventories typically rely on a large number of emitting categories, on databases mapping various source types (e.g. mobile vs. stationary sources, point, line and area sources), on emission factors estimating emission rates associated to each category and on proxies suitably performing emission spatial and temporal disaggregation (IPCC guidelines for national greenhouse gases inventories, 1996, 2006 and following corrigenda^1^). Uncertanties associated with each step in compiling emission inventories typically sum up, though it is complicated to estimate the total effect due to the difficulty in ascertaining the uncertainty at each step and how uncertainties interact with each other (Winiwarter and Muik 2010). Moreover, uncertainties may change over the years with the improvement of emission-producing activities and source characterization (Jonas et al. 2014; Lesiv et al. 2014), and their knowledge therefore becomes important for policymakers and for planning emission reduction strategies in view of the next objectives (Jonas et al. 2014). The capability of validating inventories against independent estimates is thus of great importance, as well as the development of reproducible validation methodologies that are applicable worldwide including in emerging economies.

Puff dispersion models have been widely used by the scientific community to assess pollutant dispersion and deposition, reaching a robust state of the art. Indeed, puff models have been chosen by US EPA for simulating atmospheric dispersion (EPA 2005). Puff models treat pollutant emissions according to a Lagrangian approach as a series of puffs, i.e. discrete packets of pollutant material (Scire et al. 2000b) that are influenced by advection and aging. During dispersion, puff size and concentration change following atmospheric turbulence, while pollutant concentration variation within puffs is treated through a Gaussian approach. These models have been used to simulate dispersion of a wide variety of pollutants from particulate matter (Barna and Gimson 2002; Villasenor et al. 2003; Leone et al. 2016; Holnicki et al. 2016), to gaseous pollutants such as sulfur dioxide (Elbir 2003; Abdul-Wahab et al. 2011; Holnicki et al. 2016; Calastrini et al. 2008), other organic oxides (Holnicki et al. 2016; Calastrini et al. 2008), volatile organic compounds (Holnicki et al. 2016) and even odour intensity (Vieira de Melo et al. 2012).

In order to be properly applied, puff models need the external provision of full and time-varying fields of both meteorological and micrometeorological variables over the whole domain. This level of information is usually provided by the MM5 model (Dudhia 1993), and recently by the Weather Research Forecast (WRF) model (Skamarock et al. 2008), which have become the dominant non-hydrostatic models with hundreds of academic as well as commercial users around the world. WRF, in particular, allows for the dynamical spatial and temporal downscaling of reanalysis products (Soares et al. 2012), therefore improving the performance of, for example, Lagrangian models (Bowman et al. 2013). Resolution of WRF outputs may be further improved by using the CALMET diagnostic meteorological post-processor (Scire et al. 2000a), in order to better resolve terrain topography and land use (Hernández et al. 2014). Despite the potential improvement in spatial resolution and trajectory simulation, there are still biases in computed trajectories due to non-perfect matching between meteorological transport fields and real meteorological situations (type I error), as well as due to their limited spatio-temporal resolution (type II errors) (Bowman et al. 2013). In this regard, Gioli et al. (2014b) performed a detailed comparison of the WRF/CALMET modelling chain against aircraft measurements, finding that model performance varied depending on season, land use and orography, with overall agreements ranging between 2% (inland, hilly areas) and 31% (coastal areas).

Besides dispersion models, emissions may be studied through a mass balance approach: this relies on contemporary measurements of concentrations (or densities) and atmospheric transport (i.e. wind speed and direction) in order to estimate the strength of the emitting source. The approach was initially applied to measure ammonia fluxes from small plots (Denmead et al. 1977; Wilson et al. 1982; Wilson et al. 1983), and has since been applied to different sources, also using small aircraft. The airborne approach to source emission estimation was first described in a paper by Brooks, Crawford and Oechel (Brooks et al. 1997), where a small aircraft (a Rutan Long-EZ) was flown downwind of the Prudhoe Bay oil companies in the constant flux layer (10 m above ground level). With an experimental aircraft equipped with a fast turbulence probe and CO_2_ sensors, Brooks et al. (1997) estimated an emission from the Prudhoe Bay complex four to six times higher than that reported on the basis of fuel consumption data (Jaffe et al. 1995). The potential of such a measurement platform was therefore embraced, and the technique has been applied to gaseous emissions stemming from different sources like urban (Brioude et al. 2011; Gioli et al. 2014a; O’Shea et al. 2014; Cambaliza et al. 2014), industrial (Toscano et al. 2011) and rural (Alfieri et al. 2010).

The aim of this research is to develop a framework to estimate a source emission strength through two different approaches: (i) the mass balance method and (ii) a state-of-the-art puff dispersion model chain.

For approach (i), aircraft measurements of air transport and CO_2_ densities close to a large industrial point source were used. Being a tracer gas with no significant photochemical sink, CO_2_ is an ideal compound for atmospheric mass balance experiments since it can be sampled downwind of the source with no significant alterations in its abundance.

For approach (ii), a modelling framework was implemented integrating the Weather Research Forecast (WRF-ARW) model and CALMET meteorological models, as well as the CALPUFF Lagrangian puff dispersion model. The WRF mesoscale model was run based on two different forcings, provided by the ECMWF (European Centre for Medium-Range Weather Forecasting) ERA-Interim (Dee et al. 2011) reanalysis data and the NCEP-CFSR (National Centers for Environmental Prediction - Climate Forecast System Reanalysis) (Saha et al. 2010). Furthermore, the CALMET diagnostic model was run using in situ meteorological data measured locally by the aircraft. Summarizing, the CALMET/CALPUFF models were run according to three different meteorological combinations.

Overall, this work investigated which type of atmospheric measurements combined with models are needed to estimate an unknown emission strength, assessing if simple sensors and platforms could be deployed: low-cost unmanned aerial vehicles (UAV) could, for example, be used to sample pollutant concentrations downwind without the need for concurrent measurement of the transport field. The latter would in fact be integrated by the modelling chain through large-scale meteorological forcings.

## Materials and methods

### Airborne measurements

Airborne sampling was conducted using a Sky Arrow 650, a small environmental research aircraft with a mounted Mobile Flux Platform (MFP) instrumental array. This incorporates a best available turbulence probe (BAT) (Crawford and Dobosy 1992) and an infrared gas analyzer (Li-7500, LiCor, Nebraska, USA) for molar densities of CO_2_ and H_2_O. The BAT probe measures the air velocity with respect to the aircraft by means of a nine-hole hemispheric pressure head. A GPS unit coupled with accelerometers allows both high and low frequencies of the 6-degree-of-freedom (DoF) aircraft motion to be covered, and therefore to recover the actual wind components from the measure of air velocity by subtraction. Data were collected and processed at 50-Hz frequency, while for this study they were filtered to 1 Hz by block-averaging. The aircraft platform (along with all its payload) is extensively described in Gioli et al. (2006) while Vellinga et al. (2013) details the principles of the MFP operation and the in-flight probe calibration procedure.

**Fig. 1:**
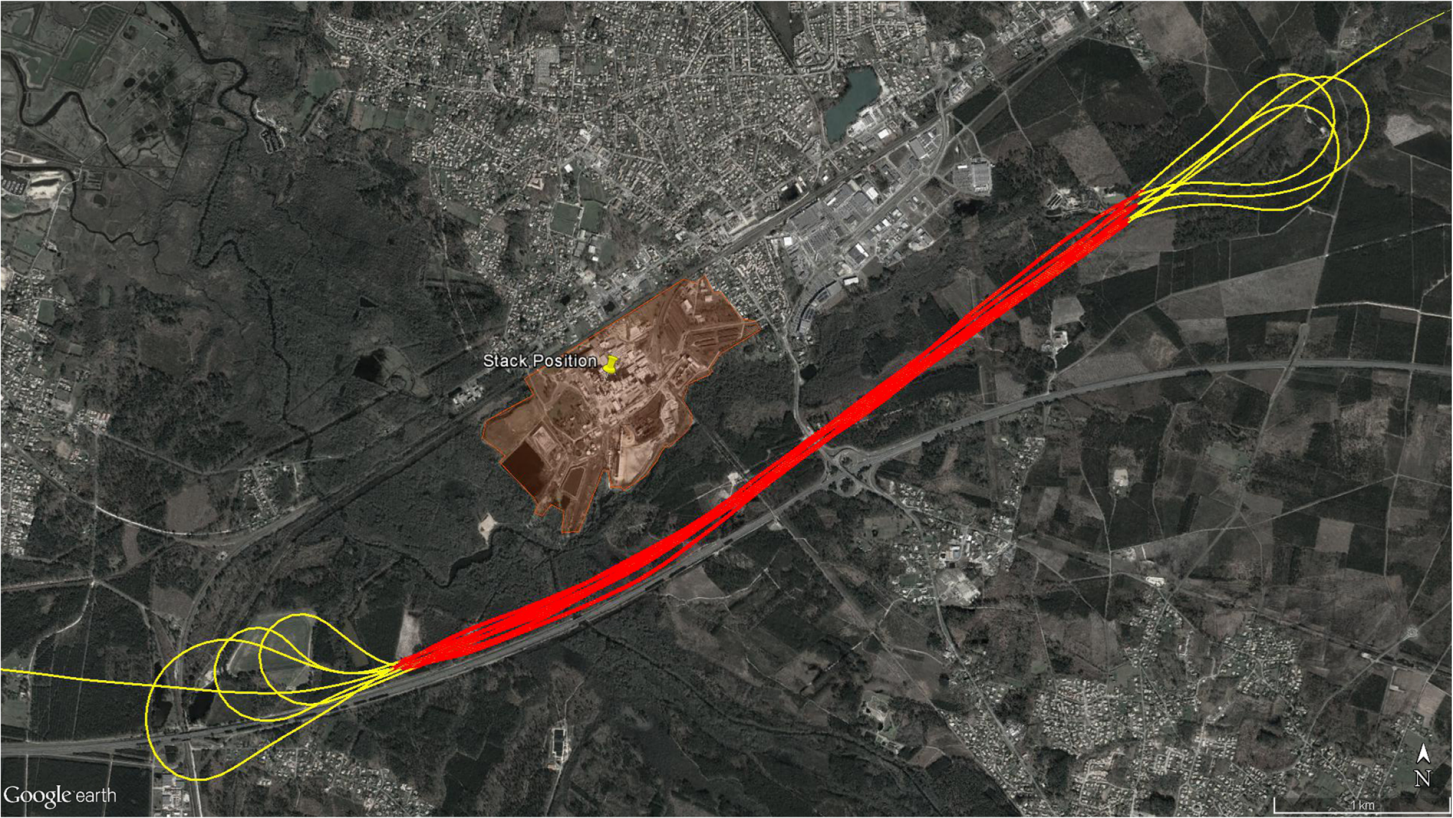
Overview of the study area. The industrial plant complex is highlighted in orange and stack position indicated by a placemark. Flight tracks, running on 28th of May 2005, are also shown: red tracks denote the flights selected for the analysis, while yellow (turning) tracks were excluded

Flights were performed on the 28th of May 2005 close to the small town of Biganos in southern France, downwind of the plants of the Smurfit Kappa industrial group (Fig. 1). Seven transects were flown downwind of the source in order to intercept the plume coming from the industrial point source. Only the straight and central sections of the flight were selected for analysis, excluding all the turns at the end of each transect.

**Fig. 2:**
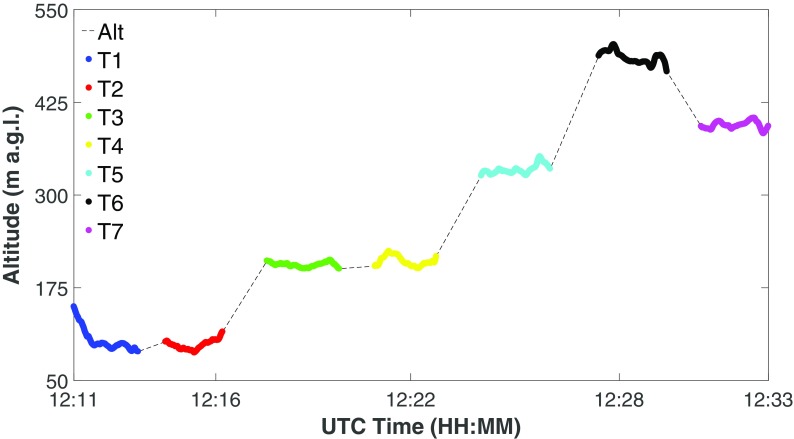
Flight vertical profile: different colours identify flight subsections pertaining to different transects. The highest transect (T6, black dots) was used to compute CO_2_ background density

The seven transects cover five height levels at which the plume is sampled: lower levels have a higher number of data in order to better resolve the emissions (Fig. 2). The average heights above ground level (a.g.l.) of the various levels are 101 m (T1 + T2), 208 m (T3 + T4), 334 m (T5), 485 m (T6), and 394 m (T7). All data from an altitude above the average altitude of the highest transect (T6), since it did not intercept the plume, were selected to compute CO_2_ background density.

### Point source details

The Smurfit-Kappa industrial complex comes under both the 2003/87/CE European Directive governing emission trading (and therefore CO_2_ emissions control) and the 166/2006 European regulation pertaining to the creation of a European pollutant release and transfer registry: CO_2_ emissions data are therefore available online (on the website of the French registry for the emissions of pollutants^2^). A total CO_2_ emission amount (from both biomass and non-biomass origins) of 973,000 t per year,corresponding to 30.8 kg s^−1^, was extracted for the year 2005.

### Aircraft mass balance

**Fig. 3:**
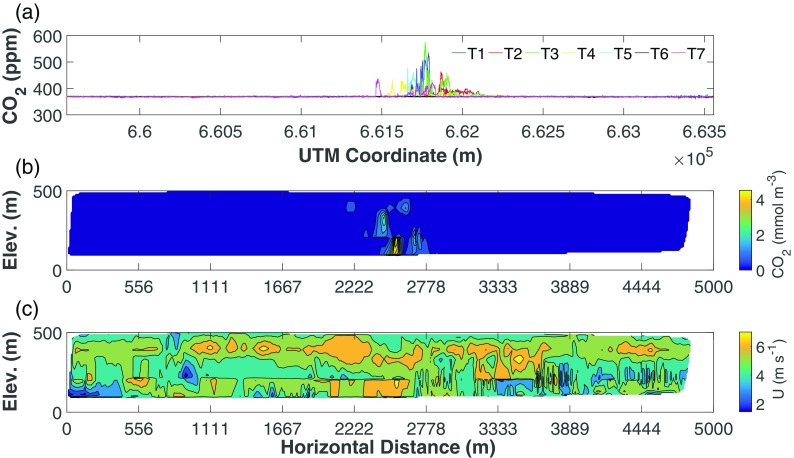
CO_2_ flux across the ideal surface *S*. Figure 3a (topmost panel) shows the aircraft concentrations over the various transects, Fig. 3b (middle panel) shows the interpolation of the aircraft data to the regular grid (after background removal and conversion to density), and Fig. 3c shows the perpendicular wind speed (U) over the interpolated domain. In **b** and **c**, Elev. indicates the vertical distance (elevation) on the interpolation grid

The CO_2_ mass balance was computed on an idealized surface *S* corresponding to the aircraft track, and extending vertically from the ground to the highest flight transect (Fig. 3). 3D position data were therefore converted into a 2D cartesian grid aligned with the aircraft track by means of a rotation matrix. The rotated wind speed (in m s^−1^) and CO_2_ density (in mmol m^−3^) were linearly interpolated on a regular grid of 10 m on the *S* surface, utilizing a scattered interpolant. The horizontal dimension ranged from 0 to 5000 m, the vertical one from 0 to 500 m, generating a grid of 51 × 501 points, with a total area of 2555.1 km^2^. The gridded interpolation output is represented in Fig. 3b.

The CO_2_ background was removed by converting molar densities to mixing ratios, subtracting the average value from the background data (Fig. 3a), and converting back to molar densities. The mass balance was then computed as the integral of the product of wind speed and CO_2_ density, obtaining a flux (in mmol m^−2^ s^−1^) across the surface *S*:

1 $$ {\mathrm{F}}_{\mathrm{C}{\mathrm{O}}_2}={\iint}_0^{\mathrm{MAX}}\left(\left[\mathrm{C}{\mathrm{O}}_2\right]\times {\mathrm{U}}_{\perp}\right)\mathrm{dxdz}{F}_{C{O}_2}={\iint}_0^{\mathrm{MAX}}\left(\left[C{O}_2\right]\times {U}_{\perp}\right) dxdz $$

where *x* and *z* represent the two dimensions of the cartesian grid aligned to *S*, *U*_⊥_ is the magnitude of the rotated wind speed perpendicular to *S* (in m s^−1^), [CO_2_] is the CO_2_ molar density (in mmol m^−3^), and ${\mathrm{F}}_{\mathrm{C}{\mathrm{O}}_2}$ is the CO_2_ flux across *S*. Given the removal of background CO_2_ and integration across the surface (i.e. MAX, the 25,551 points of the grid), ${\mathrm{F}}_{\mathrm{C}{\mathrm{O}}_2}$ represents the amount of mass advected through *S* (i.e. the mass balance of the idealized surface).

### Sensitivity analysis

The sensitivity of the mass balance estimate was tested with respect to the uncertainty in wind speed, CO_2_ density and interpolation methods following Cambaliza et al. (2014). Uncertainty in calculating wind speed and CO_2_ density was assessed by binning block-averaged wind data into 10-m altitude windows (corresponding to the interpolator altitudinal resolution) and estimating the 95% confidence intervals. These uncertanties were then propagated to the final emission estimate through the mass balance calculations (Eq. 1). Uncertainty in the interpolation methods was assessed by running the scattered interpolant according to three configurations following different interpolation algorithms: linear, natural and nearest neighbour. Since the aircraft transects did not cover all the area from the surface up to the maximum flight altitude, the impact of the not measured area was analyzed by extrapolation: wind data were extrapolated via a log-linear regression that took into account the PBL atmospheric stability, while empirical extrapolated profiles were used for CO_2_ data. Since no ground measurements were available, CO_2_ was extraploated to ground level using ordinary kriging via the Saga GIS software (Conrad et al. 2015). For this procedure, the kriging grid was set to exactly match the interpolation grid, and the kriging algorithm was set to check the 20 nearest points within a 200-grid unit range around the data points (omni-directional search around the interpolation grid). A third-degree polynomial model was then fitted to the variogram to obtain the final extrapolated data on the regular grid.

### WRF/CALMET setup and forcing

**Fig. 4:**
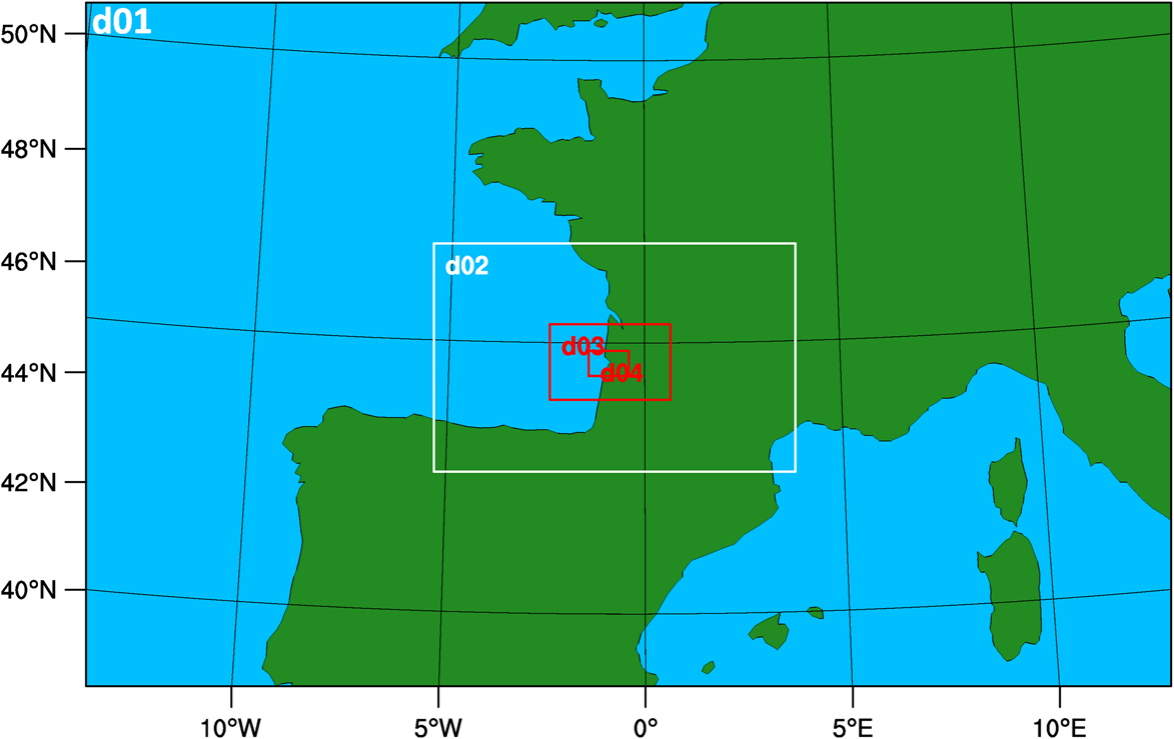
Four nested domains used in WRF model application: horizontal resolution from outermost (d01) to innermost (d04) domains are 27, 9, 3 and 1 km (1:3 nesting ratio applied)

In this study, WRF-ARW (version 3.5.1) was configured with four nested grids (Fig. 4). The outermost domain (D1) covered most of Western and Central Europe to provide the boundary conditions for the intermediate domains, D2 and D3. The D1 domain had a staggered grid size of 82 × 52 with a horizontal resolution of 27 km. Nested domains were two-way coupled, with a 1:3 grid ratio, so that the intermediate domains, D2 and D3, had horizontal resolutions of 9 and 3 km, respectively. The innermost domain, D4, encompassed the whole territory of Biganos and had 82 × 52 grid points with a horizontal resolution of 1 km. Thirty-five sigma levels were used from the ground to the top (= 50 hPa), with the first 10 layers concentrated in the lower atmosphere. The domains’ information is summarized in Table 1.

**Table 1:** Details of WRF-ARW domains applied to Biganos

Domain	Nx (cells)	Ny (cells)	∆*x* = ∆*y* (km)
D1	82	52	27
D2	82	52	9
D3	82	52	3
D4	82	52	1

Two different WRF simulations were performed over a 2-day period on the four nested domains. Initial and boundary conditions for the first simulation were provided every 6 h (at 0000, 0600, 1200 and 1800 UTC) by the ERA-Interim reanalysis data, while for the second simulation they were provided by the NCEP-CFSR data. The ERA-Interim reanalysis uses the T255 spectral method and the N128 reduced Gaussian grid (for a final resolution of around 0.7° at the equator), while the CFSR reanalysis has a spatial resolution of 0.5° for pressure-level variables and 0.3° for the surface variable (T382), and a subset from both is incorporated into the WRF-ARW pre-processor (WPS). The model was run with a 24-h spin-up time and with the parametrizations summarized in Table 2 following Mohan and Bhati (2011) and Santos-Alamillos et al. (2013).

**Table 2:** WRF-ARW parameterizations; letters indicate the appropriate literature for the given scheme

Physics module	Chosen scheme
Microphysics	Morrison double-moment scheme^a^
Longwave radiation	RRTM scheme^b^
Shortwave radiation	RRTMG scheme^c^
Surface layer	Revised MM5^d^
Land surface	Noah land surface model
Planetary boundary layer	Yonsei University scheme^e^

^a^Morrison et al. 2009

^b^Mlawer et al. 1997

^c^Iacono et al. 2008

^d^Jiménez et al. 2012

^e^Hong et al. 2006

The WRF model was coupled with CALMET (Scire et al. 2000a), version 6.5, to provide a wind field detailed estimation close to the paper factory in Biganos. CALMET uses terrain-following vertical coordinates that were set to 15 levels, spanning from 0 to 2000 m a.g.l. The D4 wind fields from the WRF prognostic model with 1-km resolution were incorporated every hour by CALMET as the initial guess wind field. The latter was then adjusted for kinematic effects of terrain, slope flows and terrain blocking effects using fine-scale terrain and land use data. Terrain data were retrieved from the Advanced Spaceborne Thermal Emission and Reflection Radiometer (ASTER) global digital elevation model (with an accuracy between 10 and 25 m^3^), while land use data were extracted from the most recent CORINE Land Cover database with a resolution of 100 m (Büttner and Kosztra 2007). In order to resolve the complex terrain structure, CALMET was configured with a high-resolution domain, which was set up with 255 × 225 grid points and a 200-m grid spacing along *x* and *y* directions.

As well as being initialized by WRF-modelled outputs, CALMET was also run with locally collected surface and profile observations. Surface observations were derived as a combination of measurements from Merignac Airport meteorological terminal aviation routine (METAR) reports (cloud cover and ceiling height), and from the LeBray eddy-covariance tower (all other variables); profile observations were directly derived from the aircraft flights. The latter model run combination was performed in order to assess whether the interpolation of 3D data coming from a coarse meteorological forcing could outperform (or not) the use of in situ, though localized, profile information. Summarizing, CALMET (and thus CALPUFF, see later) was run according to three meteorological initializations: (i) ECMWF, i.e. using WRF outputs initialized by ECMWF; (ii) CFSR, using WRF outputs initialized by CFSR, and (iii) IN SITU, using locally observed information.

### Particle transport and diffusion

Both 2D and 3D meteorological fields calculated by CALMET were used as input to the CALPUFF non-steady-state Lagrangian Gaussian puff model. CALPUFF is capable of simulating the effects of time- and space-varying meteorological conditions on pollutant transport, transformation and removal (Scire et al. 2000b). The model can accommodate arbitrarily varying emissions from point, line, area and volume sources. It is intended for use on scales from tens of meters to hundreds of kilometers away from a source. CALPUFF contains algorithms for near-source effects such as building downwash, transitional plume rise, partial plume penetration and subgrid-scale terrain interactions, as well as longer-range effects such as pollutant removal (wet scavenging or dry deposition), chemical transformation, vertical wind shear and overwater transport (Scire et al. 2000b). For the purpose of this study, three CALPUFF runs were perfomed on the same spatial and temporal domains as CALMET, i.e. ECMWF, CFSR and IN-SITU. A single emitter was located at the Biganos tall stack (660,639.5–4,943,911 UTM zone 30 N) and set to an arbitrary continuous CO_2_ emission at unit strength (i.e. 1 kg s^−1^). The stack characteristics were defined according to chimney no. 9 from the official document regarding the industrial plant^4^ closer to the aircraft measurement data: the stack had a height of 100 m, diameter of 3.5 m and a fume exit velocity (normalized to a 400 K temperature) of 7 m s^−1^. Continuous constant emission was deemed acceptable given that pulp and paper production are continuous-flow industrial processes run constantly.^5^ The model was set up in order to output densities (in mg m^−3^) at 234 fixed point receptors. The receptors were chosen in a manner to match an equivalent number of points along the aircraft tracks, allowing for a direct comparison between estimated outputs and measured data. An additional set of 702 receptors was added in the same latitudinal and longitudinal positions as the previous 234, at heights between 10 and 70 m, in order to explore the area beneath the flight tracks.

### Emission strength estimation

The ratio between the integral concentration along the aircraft points and the receptors was used to derive the multiplier needed to make simulations and measurements match (following Eq. 2). Given that the source strength was set at 1 kg s^−1^, this multiplier also represents the exact emission strength that the model would need to match the aircraft data.

2 $$ \eta =\underset{i=1}{\overset{i=N}{\int }}\frac{A_i}{M_i} $$

where *η* represents the emission strength needed for the model (*M*) to match the aircraft data (*A*). The integral is performed along the various *N* receptors (indicated by the *i* subscript).

## Results and discussion

### Dispersion modelling

**Fig. 5:**
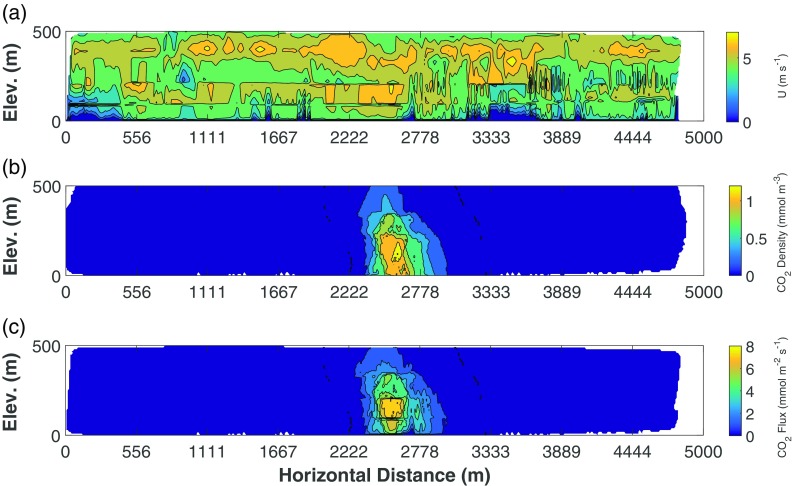
Extrapolated matrices of perpendicular wind speed (U, panel **a** on top), CO_2_ background-removed density (**b**, middle) and CO_2_ flux (**c**, bottom). In all the panels, Elev. indicates the vertical distance (elevation) on the interpolation grid

The point source emission rates provided by the inventory and estimated by the mass balance method are summarized in Table 3, along with those calculated by applying the dispersion model chain according to the three run combinations detailed in the “Materials and methods” section. Estimated average wind speed values are also reported (Fig. 5).

**Table 3:** Point source emission strength estimation based on inventorial datum, mass balance and WRF/CALMET/CALPUFF modelling chain

Run name	Data source	Method/models	Estimated emission strength		Estimated average wind speed (m s^−1^)	
			Value (kg s^−1^)	Difference vs. inventory (%)	At receptor points	Over the whole grid
	Inventory	–	30.8	0	N/A	N/A
AIRCRAFT	Aircraft obs.	Mass balance	27.4–36.2	− 11.6/+ 16	5.6 ± 1.	N/A
	Aircraft obs.	(Extrapolated) mass balance	38.3	+ 21.7	5.6 ± 1.	N/A
ECMWF	ECMWF reanalyses	WRF/CALMET/CALPUFF	36.8	+ 17.5	3.7 ± 0.2	3.04 ± 0.6
CFSR	CFSR reanalyses	WRF/CALMET/CALPUFF	48.5	+ 44.6	4.7 ± 0.6	4.21 ± 0.7
IN-SITU	Aircraft obs.	CALMET/CALPUFF	41.4	+ 29.4	5.7 ± 0.6	3.8 ± 0.7

**Fig. 6:**
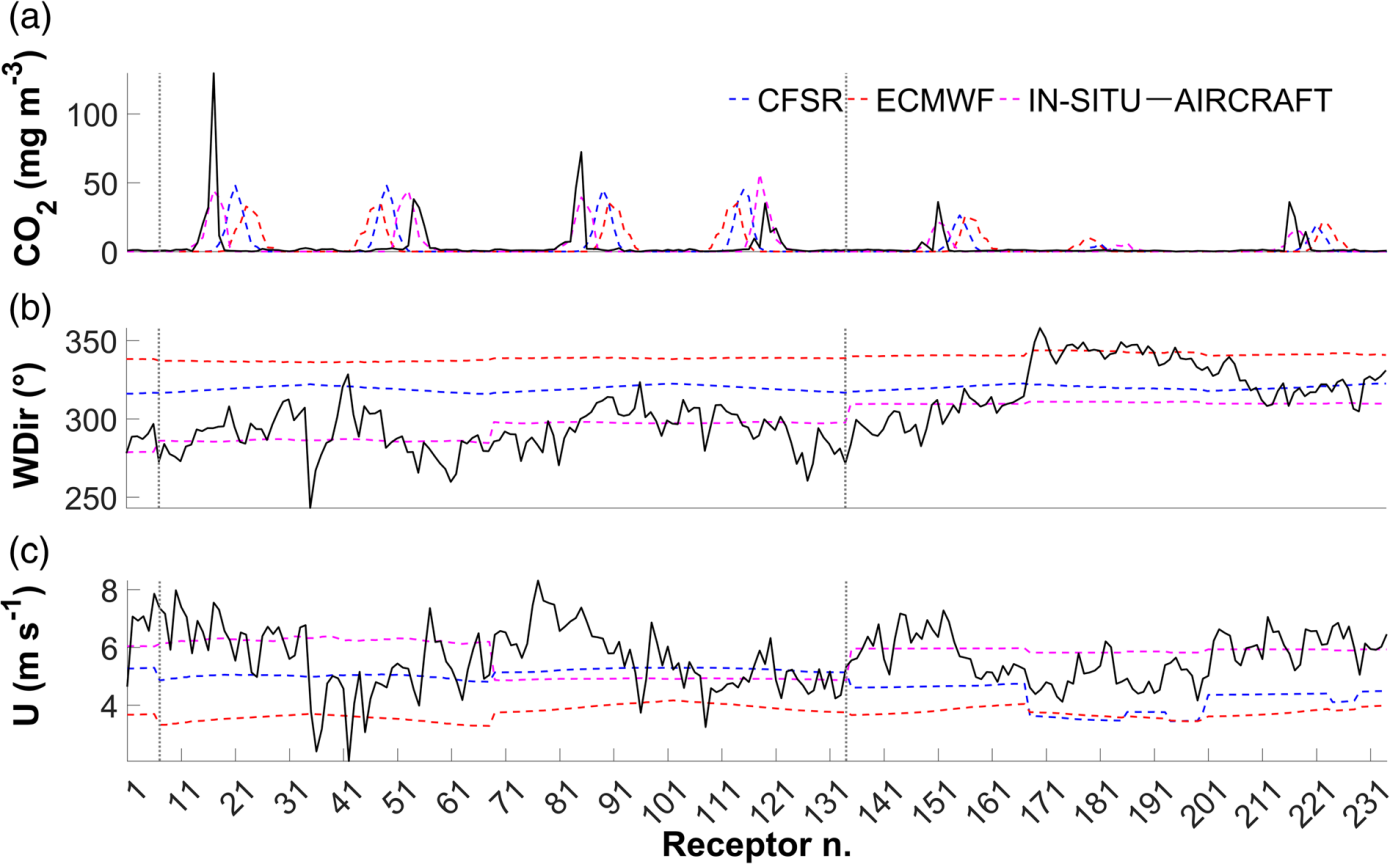
Comparison between model outputs and aircraft data on the 234 receptors for CO_2_ densities after optimization (**a**, top), wind direction (WDir, **b**, middle) and wind speed (U, **c**, bottom)

Figure 6 reports the CO_2_ density (after multiplication by the *η* coefficient; see Eq. 2 and Fig. 6a), wind direction (Fig. 6b) and speed (Fig. 6c) calculated at the 234 receptor points. In particular, Fig. 6a shows that receptors 7 to 134 (the area enclosed by the dotted grey lines in the figure, corresponding to layers 3 and 6 of the CALMET model and T1 to T4 of aircraft tracks) give the greatest contribution to the integral of the CO_2_ concentration (ranging from 71.8 to 79.6% between the various model initializations). This outcome is consistent with Fig. 3a, showing that transects T1 to T4 intercept most of the CO_2_ concentration. The dispersion model chain therefore appeared quite capable of capturing the plume’s vertical structure. Conversely, a discrepancy in the plume’s horizontal structure vs. aircraft data may be observed, as evident from the differences in wind and flight direction (the former are clearly highlighted in Fig. 6b).

Focussing on Table 3, emission strength values for ECMWF, CFSR and IN-SITU runs were obtained as unique solution of Eq. 2 after implementing an iterative process involving application of the three corresponding CALMET/CALPUFF model combinations. Consistently with the achieved results, higher wind speeds across the simulation domain correspond to lower overall concentrations requiring a higher multiplier to match aircraft concentrations (Eq. 2). This is corroborated by considering the whole modelled domain (column 7) rather than the few points matching aircraft and modelled data (column 6). Actually, dispersion modelling results were quite sensitive to changes in wind speed: all whole-grid averages are within 1.2 m s^−1^ of one another, but these differences result in estimated source strengths differing by more than 10 kg s^−1^ (considering ECMWF and CFSR runs). Even in the case of a data-based initialization (i.e. run IN-SITU), the small discrepancy introduced by the spatialization (a 1.2% difference in the receptor-derived wind speed average) was enough to make the estimated source strength change by 3.1 kg s^−1^ (considering the extrapolated mass balance which also takes into account the below-aircraft domain). In any case, the use of measured data (run IN-SITU) provides estimates that are closer to the measurements in terms of plume shape: the horizontal discrepancy seen in Fig. 6a is far less prevalent in the IN-SITU run than in ECMWF and CFSR, which is in good agreement with Fig. 6b. The latter shows wind direction patterns across the 234 receptors for all model combinations,with IN-SITU being the run with the smallest RMSE (17.4° vs. 26.1° for CFSR and 40.5° for ECMWF).

Considering all CALMET/CALPUFF model runs, ECMWF proved to be best at reproducing the inventorial datum (17.6% overestimation). Remarkably, this dispersion run using only modelled data as meteorological initialization performed better than the one (IN-SITU) using aircraft meteorological observations (29.4% overestimation). Clearly, the differences found in the dispersion model performances should be ascribed to differences resulting from the meteorological section of the model chain. Not only do the achieved outcomes therefore highlight the importance of relying on reliable in situ meteorological observations, but also that using different meteorological forcings may lead to substantial differences.

Angevine et al. (2014) did an analogous investigation by initializing the FLEXPART Lagrangian dispersion particle model (Brioude et al. 2013) with different WRF-ARW configurations, including two forcings (Global Forecast System and ERA Interim). Among the various thorough comparisons, they investigated the differences between modelled CO tracer dispersal and measured CO from aircraft flights. One of their conclusions was that for single-mesoscale Lagrangian simulations, the uncertainty for passive tracers ranged between 20% (in favorable situations) and 60% (in unfavorable situations): these results are quite comparable with the uncertainty we observed with our CALPUFF simulations, as the percentage differences between inventorial data and the various methods ranged between − 11.6 and 48.6% (Table 3). Bowman et al. (2013) made suggestions that could potentially reduce these transport field-related uncertainties, and two that are particularly relevant are (i) improving the output of the global circulation models that are used as input for mesoscale meteorological modelling (such as increasing the temporal frequency of outputs, inserting information about subgrid-scale processes) and (ii) introducing some modifications to the mesoscale transport field that are finally used as input to a dispersion model (again increasing the temporal frequency of outputs and time average winds between output intervals to improve accuracy of trajectories). Besides considering the uncertainties within the models themselves, attention must be paid when comparing numerical models with aircraft-measured variables. Models based on Reynolds-averaged Navier–Stokes (RANS) equations, in fact, provide results representative of space and time averages of physical variables. The effective space and time resolution of WRF and CALPUFF depends on the computational domain grid spacing, implicitly assuming an averaging time window large enough to sample the whole boundary layer turbulence spectrum. Instead, airborne measurements represent short temporal scales, and are individually affected by instantaneous turbulent eddies, especially in neutral to convective conditions: at an average ground speed of 40 m s^−1^ and a 50-Hz frequency, the aircraft is in fact able to measure at a 0.8-m resolution, meaning that it can sample small and transient turbulent eddies, which are “invisible” to the models. We should therefore expect observations to include short-wavelength fluctuations and possibly transient structures that cannot be reproduced by any numerical model simulation. To overcome this issue, multiple aircraft passes were made at each altitude and averaged to reduce the influence of high-frequency turbulent fluctuations.

### Mass balance

The flight transects at various altitudes clearly revealed the CO_2_ plume generated by the industrial stack (Fig. 3a). CO_2_ concentrations reached the highest values at the T3 transect, followed by the lower transects (T1 and T2). The highest transect T6, since it did not intercept the plume at all, was chosen as the base for calculating the background value. The horizontal spread of the measured plume along the flight tracks, at a distance of 1000 m downwind of the stack, was 840 m (with the highest peaks in the central part of the plume), while outside the plume the CO_2_ concentrations were basically constant throughout with background values equal to those measured at the highest elevation in T6 (Fig. 3a).

Gridded values of CO_2_ fluxes and wind speed on the *S* plane are shown in Fig. 3b, c. The wind component perpendicular to *S* did not reveal a relevant vertical variation (Fig. 3c), with a mean of 5.0 ± 0.1 m s^−1^. Such a significant wind speed magnitude across the computational domain was of paramount importance: as noted in the pioneering studies of Denmead et al. (1998) up to the more recent experiments of Gioli et al. (2014a), too weak winds may adversely affect the mass balance computation due to a decrease in stationarity.

The computed mass balance resulted in a predicted source strength of 31.8 kg s^−1^ (a summary of computations is given in Table 4).

**Table 4:** Mass balance results and emission strength estimate. The effective area indicates the total surface area where cells were filled by the interpolation procedure (in certain areas, for example below the aircraft minimum altitude, no data were present)

Effective area (km^2^)	Average CO_2_ flux (mmol m^−2^ s^−1^)	Raw emission strength (kg s^−1^)	Wind speed uncertainty (%)	CO_2_ density uncertainty (%)	Interpolation uncertainty (%)	Sensitivity-bounded emission strength (kg s^−1^)
1926.4	0.37	31.8	12.1	0.10	1.6	27.5–36.2

The difference between estimated emission strength and overall inventorial yearly amount was equal to 1.0 kg s^−1^, equivalent to 3.2%.

The overall mass balance uncertainty results from a combination of uncertainty in wind speed and CO_2_ density measurements. The 95% confidence limit of altitude-binned wind speed was ≈ 0.3 m s^−1^, which, added to the instrumental uncertainty of the BAT probe (which was estimated by Garman (2009) to be around 0.4 m s^−1^), resulted in a total uncertainty of 0.7 m s^−1^. The propagation of wind speed uncertainty produced a percentage variation of the emission rate of ± 12.1%. Both the uncertainty in wind speed and its propagation to emission rates were comparable with Cambaliza et al. (2014). Given the effect that wind speed has on the mass balance and that the measurement uncertainty was close to the instrumental one, the great importance of correctly calibrating the flux platform before each flight is clear. Mean uncertainty in CO_2_ density was ≈ 0.03 mmol m^−3^, and the corresponing percentage change in the emission estimate after error propagation was 0.1%. The combined uncertainty is reported in absolute terms in Table 3, where the net effect of the two methods used for the interpolation is also reported.

The effect of data extrapolation from the minimum flight altitude down to the ground (Fig. 5a–c) shows that a significant part of the plume could have been omitted from direct measurements (Fig. 5b, c), taking into account that the extra density evaluated by the ordinary kriging procedure would increase the mass balance up to 38.3 kg s^−1^ (an 18.5% difference from what was found with simple linear interpolation).

When measuring large area emissions with a mass balance method, determination of the bounding volume (and, therefore, PBL height) becomes a critical factor, also driving the mass budget uncertainties (Alfieri et al. 2010; Gioli et al. 2014a). However, the good correspondence of the mass balance calculations with the inventorial data (and the disappearance of significant concentration peaks at the highest flight transects) showed that the Biganos plant offered a simple enough situation, where the distinction between the source’s plume and background levels required no assumptions about PBL structure and source variability. The Biganos sampling approach is corroborated by Denmead (2008) who states that for sources of limited upwind size, a downwind sampling can suffice, provided that the emission is sampled along its vertical extent and that background concentrations are either known or measurable. In fact, mass balance methods tend to perform better when there is a certain difference between source and background (Denmead 2008; Loh et al. 2009) and in the case of Biganos there is a 30% difference between the measured plume peak (493.5 ppm) and background value (368.8 ppm), which is well above the suggested 1% difference for line-averaged gas measurements (Loh et al. 2009). Mass balance methods should, in fact, rely on spatialized measurements (such as line measurements) since they are insensitive to lateral displacement (Flesch et al. 2004) and maximize useable wind directions (Loh et al. 2009): the multi-transect aircraft sampling that has been used in the present work agrees well with all the aforementioned necessities.

## Conclusions

In this work, two methods were used to estimate the emission strength of an industrial point source of CO_2_ emissions, which is located in Biganos, France: (i) the mass balance method, based on aircraft observed data, and (ii) a dispersion modelling framework, integrating the CALMET diagnostic meteorological model and CALPUFF puff dispersion model. In particular, the CALMET/CALPUFF model chain was run according to three meteorological initializations: (i) WRF-modelled outputs initialized by ECMWF reanalyses; (ii) WRF-modelled outputs initialized by CFSR reanalyses and (iii) local in situ observations. Government inventorial data were used as reference for all applications. The two approaches compared resulted in both advantages and weaknesses, which make an integrated framework particularly interesting.

The mass balance approach is capable of capturing the point source emission strength provided that measurements are made based on stationary conditions, above a minimum wind speed, and that the meteorological variability and PBL height can be correctly sampled. Indeed, the mass balance reproduces a snapshot of the actual emission scenario: while this allows a constant emission rate such as the one from a continuously emitting production plant to be estimated, it only gives instantaneous and precisely located information on the emission source. It must be borne in mind that aircraft measurements are expensive, subject to favourable weather conditions and strictly localized in time and space.

Conversely, all the above limitations are generally overcome by a dispersion model, which is capable of reproducing not only the change in the emission rate over time, but also the 3D time-dependent plume structure and its final fate in the atmosphere. A winning strategy was the use of meteorological reanalyses in place of locally observed data: in particular, the ECMWF meteorological forcing which passed through the WRF mesoscale model returned an emission strength estimation only slightly higher than the one achieved applying the mass balance to airborne measurements. The clear added value of the modelling approach is its capability of estimating the source emission rate *whenever and wherever*, not just over *that* time frame and *that* geographical location as observed by the aircraft. Furthermore, if properly set up and initialized, a similar modelling approach might be more useful to researchers and regulatory planners than the emission inventories themselves, as it provides an overall emission assessment that, unlike the latter, can take into due account any dynamically varying operative source conditions.

However, the dispersion modelling approach proved to be highly sensitive to the meteorological data source used as initialization, which strongly affected the modelled plume shape and trajectory. Particular care should be taken when defining model setup and forcing, initial and boundary conditions and point source characteristics. In any case, the importance of estimating uncertainties and errors in the meteorological inputs should be stressed. Strategies for applying a dispersion model to the situation described in this paper would therefore include a comparison of modelled meteorological fields with observations or usage of ensemble simulations.

Both the mass balance and dispersion modelling framework deployed in the current work were applied on CO_2_, an inert gas. It is therefore important to emphasize that both methodological approaches might easily be extended to other inert compounds typically emitted by a point source, i.e. SO_2_, CO, primary PM_10_, heavy metals, etc. This gives new insights into the validation of currently developed emission inventories, not only in assessing their emission rates, but also in reliably reproducing their variation over time (e.g. by hour in the day, day of the week, month of the year): the latter is a typical drawback of most national emission inventories, basically designed to provide overall yearly amounts rather than 1-h varying estimates.

In the future, especially with the very fast development of small airborne platforms such as UAVs, the downwind measurement would become even simpler and cheaper if a good source emission strength estimation could be achieved. This application thus would become an interesting tool for inventory validation for both regulatory and third-party actors. While the main focus of this paper was on the estimation of point source CO_2_ emissions, recent advances in miniaturized sensors will make small UAVs capable of measuring not only air turbulence (Martin et al. 2014; Wildmann et al. 2014), but also concentrations of gas compounds other than CO_2_ (Refaat et al. 2013; Illingworth et al. 2014).

## Footnotes

1 IPCC Guidelines are available at http://www.ipcc-nggip.iges.or.jp/public/2006gl/index.html.

2 Smurfit Kappa iREP data available at http://www.irep.ecologie.gouv.fr/IREP/index.php?adr=http://www.irep.ecologie.gouv.fr/IREP/resultEtab.php?nomEtablissement=smurfit%20kappa

3 ASTER GDEM is a product of NASA and METI (10.5067/ASTER/ASTGTM.002).

4 Smurfit-Kappa installation documentation is available at http://documents.installationsclassees.developpement-durable.gouv.fr/commun/B/6/6cfce038afd44d2a9d95ce2840685c86.pdf.

5 Information about the industrial process is available at http://www.smurfitkappa.com/vHome/fr/CelluloseduPin/Pages/Presentation.aspx.
